# Supplementary material for: Water Quality and Mortality from Coronary Artery Disease in Sardinia: A Geospatial Analysis
Source: Nutrients. 2021 Aug 20;13(8):2858. doi: 10.3390/nu13082858 (PMC8399079; doi:10.3390/nu13082858)
Supplement: Supplementary file 1 [file nutrients-13-02858-s001.zip › nutrients-1303764-supplementary.pdf]

Ranges of physico-chemical parameters of waters in three Sardinian subregions

	**Nurra**	**Sulcis-Iglesiente**	**Quirra**
SMR for IHD	0.862	0.832	0,862
	**Range**	**Range**	**Range**
Total dissolved solids	230 – 1890	262 – 1463	189 – 458
pH	6.4 – 8.1	6.8 – 7.5	6.6 – 7.0
Sodium (Na^+)	30.6 – 250.0	25.0 – 56.0	11.0 – 40.0
Potassium (K^+)	1.2 – 48.3	1.4 – 25.0	0.7 – 2.3
Calcium (Ca^{2+})	28.9 – 213.0	9.0 – 91.5	0.2 – 103.0
Magnesium (Mg^{2+})	3.5 – 66.0	6.0 – 36.0	4.3 – 63.0
Bicarbonate (HCO_3^-)	19.0 – 3155.0	43.0 – 927.0	60.0 – 611.0
Sulphate (SO_4^{2-})	10.0 – 436.6	17.0 – 59.9	8.8 – 54.0
Chloride (Cl^-)	55.0 – 428.0	43.0 – 329.0	17.0 – 77.0
